# Identification of SOX2 as a novel glioma-associated antigen and potential target for T cell-based immunotherapy

**DOI:** 10.1038/sj.bjc.6603802

**Published:** 2007-06-12

**Authors:** M Schmitz, A Temme, V Senner, R Ebner, S Schwind, S Stevanovic, R Wehner, G Schackert, H K Schackert, M Fussel, M Bachmann, E P Rieber, B Weigle

**Correction to:** *British Journal of Cancer* (2007) **96**, 1293–1301. doi:10.1038/6603696

In the abstract of the above paper, a peptide with correct sequence (TLMKKDKYTL) is mentioned. This sequence should also appear in Table 1 as the sequence of peptide 60031. Unfortunately, an incorrect sequence (ALSPASSRSV) was included in Table 1 (all other data in the respective line of the table are correct and refer to peptide TLMKKDKYTL).

The corrected Table 1 is shown below.

## Figures and Tables

**Table 1:** Prediction of HLA-A^*^0201-restricted SOX2-derived peptides and determination of binding affinities by a competition assay

**Peptide**	**Position^a^**	**Sequence**	**MW**	**Length^b^**	**Score**	**rBA (%)^c^**
60029	275–283	SMYLPGAEV	965.5	9	26	97.2
60030	131–139	LLAPGGNSM	858.4	9	25	80.0
60031	118–127	TLMKKDKYTL	1239.7	10	24	43.3

MW=molecular weight; rBA=relative binding affinity.

a The given numbers indicate the position of the peptide in the amino-acid sequence of SOX2.

b Number of amino acids.

c The relative binding affinities were determined by comparing the inhibition of the reporter peptide binding by the analysed peptides in relation to the inhibition obtained with a positive control peptide, which was set as 100%. The positive control peptide was YLLPAIVHI from RNA helicase p72 and the reporter peptide was ILK(FITC)EPVHGV from HIV-1 reverse transcriptase. All peptides were used at a concentration of 10 *μ*M.

